# 2-Amino-4-(3,4-dimeth­oxy­phen­yl)-5,6-dihydro­benzo[*h*]quinoline-3-carbo­nitrile–3-amino-1-(3,4-dimeth­oxy­phen­yl)-9,10-dihydro­phenanthrene-2,4-dicarbonitrile (1/19)

**DOI:** 10.1107/S1600536811040517

**Published:** 2011-10-08

**Authors:** Abdullah M. Asiri, Abdulrahman O. Al-Youbi, Hassan M. Faidallah, Seik Weng Ng

**Affiliations:** a Chemistry Department, Faculty of Science, King Abdulaziz University, PO Box 80203 Jeddah, Saudi Arabia; b Center of Excellence for Advanced Materials Research, King Abdulaziz University, PO Box 80203 Jeddah, Saudi Arabia; c Department of Chemistry, University of Malaya, 50603 Kuala Lumpur, Malaysia

## Abstract

The asymmetric unit of the 1:19 title co-crystal of 2-amino-4-(3,4-dimeth­oxy­phen­yl)-5,6-dihydro­benzo[*h*]quinoline-3-carbo­nitrile and 3-amino-1-(3,4-dimeth­oxy­phen­yl)-9,10-dihydro­phenanthrene-2,4-dicarbonitrile, 0.05C_22_H_19_N_3_O_2_·0.95C_24_H_19_N_3_O_2_, has the atoms of the fused-ring system and those of the amino, cyano and dimeth­oxy­phenyl substitutents overlapped. The fused-ring system is buckled owing to the ethyl­ene linkage in the central ring with the two flanking aromatic rings being twisted by 31.9 (1)°. The ring of the dimeth­oxy­phenyl substituent is twisted by 72.4 (1)° relative to the amino- and cyano-bearing aromatic ring. In the crystal, mol­ecules are linked by duplex amine N—H⋯O(meth­oxy) hydrogen bonds in a cyclic association [graph-set *R*_2_^2^(7)], generating a helical chain structure extending along [201].

## Related literature

For a similar co-crystal, see: Asiri *et al.* (2011 ▶). For graph-set analysis, see: Etter *et al.* (1990 ▶).
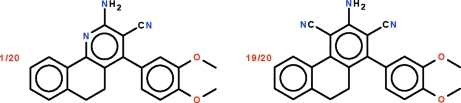


## Experimental

### 

#### Crystal data

0.05C_22_H_19_N_3_O_2_·0.95C_24_H_19_N_3_O_2_
*M* *_r_* = 380.22
Monoclinic, 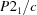

*a* = 8.9347 (3) Å
*b* = 14.4915 (5) Å
*c* = 14.7818 (6) Å
β = 103.446 (4)°
*V* = 1861.45 (12) Å^3^
*Z* = 4
Mo *K*α radiation
μ = 0.09 mm^−1^
*T* = 100 K
0.30 × 0.25 × 0.20 mm

#### Data collection

Agilent SuperNova Dual diffractometer with an Atlas detector
Absorption correction: multi-scan (*CrysAlis PRO*; Agilent, 2010 ▶) *T*_min_ = 0.974, *T*_max_ = 0.983
9240 measured reflections
4160 independent reflections
3146 reflections with *I* > 2σ(*I*)
*R*_int_ = 0.031

#### Refinement

*R*[*F* ^2^ > 2σ(*F* ^2^)] = 0.049
*wR*(*F* ^2^) = 0.124
*S* = 1.04
4160 reflections
270 parameters
H atoms treated by a mixture of independent and constrained refinement
Δρ_max_ = 0.30 e Å^−3^
Δρ_min_ = −0.24 e Å^−3^

### 

Data collection: *CrysAlis PRO* (Agilent, 2010 ▶); cell refinement: *CrysAlis PRO*; data reduction: *CrysAlis PRO*; program(s) used to solve structure: *SHELXS97* (Sheldrick, 2008 ▶); program(s) used to refine structure: *SHELXL97* (Sheldrick, 2008 ▶); molecular graphics: *X-SEED* (Barbour, 2001 ▶); software used to prepare material for publication: *publCIF* (Westrip, 2010 ▶).

## Supplementary Material

Crystal structure: contains datablock(s) global, I. DOI: 10.1107/S1600536811040517/zs2146sup1.cif

Structure factors: contains datablock(s) I. DOI: 10.1107/S1600536811040517/zs2146Isup2.hkl

Supplementary material file. DOI: 10.1107/S1600536811040517/zs2146Isup3.cml

Additional supplementary materials:  crystallographic information; 3D view; checkCIF report

## Figures and Tables

**Table 1:** Hydrogen-bond geometry (Å, °)

*D*—H⋯*A*	*D*—H	H⋯*A*	*D*⋯*A*	*D*—H⋯*A*
N3—H1⋯O1^i^	0.95 (2)	2.24 (2)	2.927 (2)	129 (2)
N3—H2⋯O2^i^	0.92 (2)	2.25 (2)	2.987 (2)	136 (2)

Symmetry code: (i) 
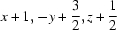
.

**Fig. 1.**
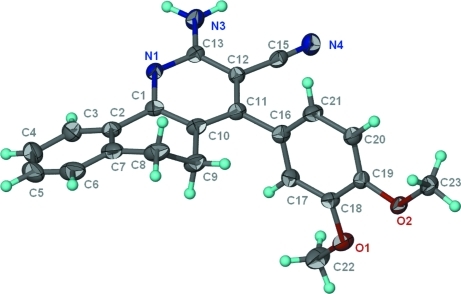
Thermal ellipsoid plot (Barbour, 2001) of C22H19N3O2 (molecule 1) at the 70% probability level. Hydrogen atoms are drawn as spheres of arbitrary radius.

**Fig. 2.**
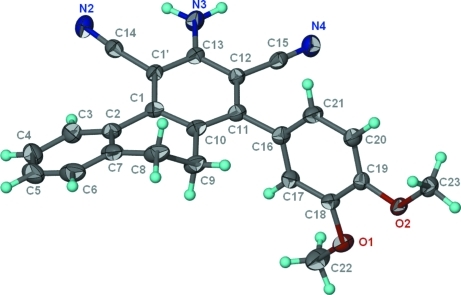
Thermal ellipsoid plot (Barbour, 2001) of C24H19N3O2 (molecule 2) at the 70% probability level. Hydrogen atoms are drawn as spheres of arbitrary radius.

## supplementary crystallographic information

### Comment

2-Amino-5,6-dihydro-4-phenyl-benzoquinoline-3-carbonitrile is synthesized from the reaction of the α-substituted cinnamonitrile, C_6_H_5_CH═C(CN)_2_, with α-tetralone in a reaction that is catalyzed by ammonium acetate. The synthesis when conducted under microwave irradiation leads to an improved yield. In previous studies, we obtained instead di-carbonitrile substituted dihydrophenanthrenes (3-amino-1-(4-methoxyphenyl)-9,10-dihydrophenanthrene-2,4-dicarbonitrile and 3-amino-1-(2*H*-1,3-benzodioxol-5-yl)-9,10-dihydrophenanthrene-2,4-dicarbonitrile) with 4-methoxybenzaldehyde and piperonaldehyde in syntheses that differed slightly from the reported ones as we used substituted benzaldehydes, α-tetralone and ethyl cyanoacetate along with a molar excess of ammonium acetate.

In this study, the reaction of 3,4-dimethoxybenzaldehyde, α-tetralone and ethyl cyanoacetate yielded the co-crystal of 2-amino-4-(3,4-dimethoxyphenyl)-5,6-dihydrobenzoquinoline-3-carbonitrile (C_22_H_19_N_3_O_2_) and 3-amino-1-(3,4-dimethoxyphenyl)-9,10-dihydrophenanthrene-2,4-dicarbonitrile (C_24_H_19_N_3_O_2_) with the components present in a 1: 19 molar ratio (Scheme I). The fused-ring system is buckled owing to the ethylene linkage in the central ring with the two flanking aromatic rings twisted by 31.9 (1) °. Relative to the amino- and cyano-bearing aromatic ring, the benzene ring is twisted by 72.4 (1) ° (Figs. 1 and 2). Molecules are linked by duplex amine N–H···O (methoxy) hydrogen bonds (Table 1) in a cyclic association [graph set *R*^2^_2_(7) (Etter *et al.*, 1990)], generating a helical chain structure extending along [2 0 1].

### Experimental

A mixture of 3,4-dimethoxybenzaldehyde (1.66 g,10 mmol), α-tetralone (1.46 g, 10 mmol), ethyl cyanoacetate (1.13 g, 10 mmol) and ammonium acetate (6.16 g, 80 mmol) in absolute ethanol (50 ml) was refluxed for 6 h. The mixture was allowed to cool and the precipitate that formed was filtered, washed with water, dried and recrystallized from DMF.

### Refinement

Carbon-bound H-atoms were placed in calculated positions [C–H = 0.95–0.99 Å; *U*_iso_(H) 1.2–1.5*U*_eq_(C)] and were included in the refinement in the riding model approximation. The amino H-atoms were located in a difference Fourier map and were refined without restraint, including their temperature isotropic displacement parameters. The compound is a co-crystal of 2-amino-4-(3,4-dimethoxy)-5,6-dihydrobenzoquinoline-3-carbonitrile (C_22_H_19_N_3_O_2_) and 3-amino-1-(3,4-dimethoxyphenyl)-9,10-dihydrophenanthrene-2,4-dicarbonitrile (C_24_H_19_N_3_O_2_). The first component is a dihydrobenzoquinoline and has only one cyano substituent. The second component is a dihydrophenanthrene with two cyano substituents. The two-coordinate N atom of the first molecule occupies the same site as the three-coordinate C atom of the second molecule. As the occupancy refined to an almost 1:19 ratio, the occupancy was then fixed as this ratio.

### Crystal data

**Table:**

0.05C_22_H_19_N_3_O_2_·0.95C_24_H_19_N_3_O_2_	*F*(000) = 797.6
*M**_r_* = 380.22	*D*_x_ = 1.357 Mg m^−^^3^
Monoclinic, *P*2_1_/*c*	Mo *K*α radiation, λ = 0.71073 Å
Hall symbol: -P 2ybc	Cell parameters from 3220 reflections
*a* = 8.9347 (3) Å	θ = 2.3–29.2°
*b* = 14.4915 (5) Å	µ = 0.09 mm^−^^1^
*c* = 14.7818 (6) Å	*T* = 100 K
β = 103.446 (4)°	Block, orange
*V* = 1861.45 (12) Å^3^	0.30 × 0.25 × 0.20 mm
*Z* = 4	

### Data collection

**Table:**

Agilent SuperNova Dual diffractometer with an Atlas detector	4160 independent reflections
Radiation source: SuperNova (Mo) X-ray Source	3146 reflections with *I* > 2σ(*I*)
Mirror	*R*_int_ = 0.031
Detector resolution: 10.4041 pixels mm^-1^	θ_max_ = 27.5°, θ_min_ = 2.3°
ω scans	*h* = −11→8
Absorption correction: multi-scan (*CrysAlis PRO*; Agilent, 2010)	*k* = −18→17
*T*_min_ = 0.974, *T*_max_ = 0.983	*l* = −19→17
9240 measured reflections	

### Refinement

**Table:**

Refinement on *F*^2^	Primary atom site location: structure-invariant direct methods
Least-squares matrix: full	Secondary atom site location: difference Fourier map
*R*[*F*^2^ > 2σ(*F*^2^)] = 0.049	Hydrogen site location: inferred from neighbouring sites
*wR*(*F*^2^) = 0.124	H atoms treated by a mixture of independent and constrained refinement
*S* = 1.04	*w* = 1/[σ^2^(*F*_o_^2^) + (0.0502*P*)^2^ + 0.6446*P*] where *P* = (*F*_o_^2^ + 2*F*_c_^2^)/3
4160 reflections	(Δ/σ)_max_ = 0.001
270 parameters	Δρ_max_ = 0.30 e Å^−^^3^
0 restraints	Δρ_min_ = −0.24 e Å^−^^3^

### Fractional atomic coordinates and isotropic or equivalent isotropic displacement parameters (Å^2^)

**Table:**

	*x*	*y*	*z*	*U*_iso_*/*U*_eq_	Occ. (<1)
O1	0.30588 (13)	0.63995 (9)	0.10804 (8)	0.0271 (3)	
O2	0.07648 (12)	0.69798 (8)	0.17421 (8)	0.0220 (3)	
N1	0.94620 (18)	0.95400 (11)	0.33260 (11)	0.0197 (3)	0.05
C1'	0.94620 (18)	0.95400 (11)	0.33260 (11)	0.0197 (3)	0.95
N2	1.21403 (18)	1.02734 (11)	0.39566 (11)	0.0279 (4)	0.95
N3	1.00292 (18)	0.87635 (11)	0.48043 (11)	0.0271 (4)	
H1	1.104 (3)	0.9012 (16)	0.4948 (16)	0.053 (7)*	
H2	0.973 (2)	0.8423 (15)	0.5258 (15)	0.035 (6)*	
N4	0.68515 (17)	0.74165 (11)	0.49326 (11)	0.0294 (4)	
C1	0.84721 (19)	0.97189 (11)	0.24628 (11)	0.0209 (4)	
C2	0.8945 (2)	1.02681 (12)	0.17292 (12)	0.0253 (4)	
C3	1.0457 (2)	1.02878 (13)	0.16227 (13)	0.0315 (4)	
H3	1.1232	0.9947	0.2038	0.038*	
C4	1.0837 (3)	1.07998 (14)	0.09164 (14)	0.0374 (5)	
H4	1.1867	1.0808	0.0847	0.045*	
C5	0.9711 (3)	1.12980 (14)	0.03145 (14)	0.0425 (5)	
H5	0.9976	1.1660	−0.0161	0.051*	
C6	0.8200 (3)	1.12739 (13)	0.03999 (13)	0.0382 (5)	
H6	0.7435	1.1618	−0.0019	0.046*	
C7	0.7794 (2)	1.07502 (12)	0.10951 (12)	0.0295 (4)	
C8	0.6159 (2)	1.06430 (13)	0.11720 (13)	0.0326 (4)	
H8A	0.5449	1.0839	0.0585	0.039*	
H8B	0.5970	1.1035	0.1682	0.039*	
C9	0.5876 (2)	0.96303 (13)	0.13680 (12)	0.0292 (4)	
H9A	0.4801	0.9547	0.1424	0.035*	
H9B	0.6036	0.9242	0.0848	0.035*	
C10	0.69749 (19)	0.93394 (12)	0.22619 (12)	0.0231 (4)	
C11	0.65552 (18)	0.87253 (11)	0.28827 (11)	0.0213 (4)	
C12	0.75689 (18)	0.85343 (11)	0.37374 (11)	0.0193 (3)	
C13	0.90408 (18)	0.89474 (11)	0.39830 (11)	0.0196 (3)	
C14	1.0941 (2)	0.99715 (12)	0.36327 (12)	0.0225 (4)	0.95
C15	0.71436 (18)	0.79079 (12)	0.43878 (12)	0.0224 (4)	
C16	0.50312 (18)	0.82492 (12)	0.26343 (11)	0.0215 (4)	
C17	0.47860 (19)	0.75520 (12)	0.19617 (12)	0.0224 (4)	
H17	0.5599	0.7375	0.1683	0.027*	
C18	0.33741 (18)	0.71173 (11)	0.16982 (11)	0.0203 (4)	
C19	0.21512 (18)	0.74044 (11)	0.20790 (11)	0.0193 (3)	
C20	0.2414 (2)	0.80721 (12)	0.27631 (12)	0.0249 (4)	
H20	0.1605	0.8251	0.3044	0.030*	
C21	0.3855 (2)	0.84870 (13)	0.30454 (12)	0.0262 (4)	
H21	0.4026	0.8937	0.3526	0.031*	
C22	0.4368 (2)	0.59651 (14)	0.08517 (15)	0.0368 (5)	
H22A	0.4020	0.5462	0.0409	0.055*	
H22B	0.5043	0.5716	0.1418	0.055*	
H22C	0.4934	0.6420	0.0571	0.055*	
C23	−0.05031 (19)	0.73108 (12)	0.20937 (13)	0.0260 (4)	
H23A	−0.1430	0.6957	0.1814	0.039*	
H23B	−0.0678	0.7965	0.1937	0.039*	
H23C	−0.0268	0.7236	0.2771	0.039*	

### Atomic displacement parameters (Å^2^)

**Table:**

	*U*^11^	*U*^22^	*U*^33^	*U*^12^	*U*^13^	*U*^23^
O1	0.0180 (6)	0.0316 (7)	0.0312 (7)	0.0003 (5)	0.0050 (5)	−0.0126 (6)
O2	0.0152 (6)	0.0253 (6)	0.0258 (6)	−0.0021 (5)	0.0051 (5)	−0.0061 (5)
N1	0.0175 (8)	0.0197 (8)	0.0218 (8)	−0.0008 (7)	0.0042 (7)	−0.0027 (7)
C1'	0.0175 (8)	0.0197 (8)	0.0218 (8)	−0.0008 (7)	0.0042 (7)	−0.0027 (7)
N2	0.0214 (8)	0.0270 (8)	0.0354 (9)	−0.0022 (7)	0.0065 (7)	0.0011 (7)
N3	0.0209 (8)	0.0325 (9)	0.0240 (8)	−0.0045 (7)	−0.0028 (6)	0.0063 (7)
N4	0.0257 (8)	0.0309 (8)	0.0315 (8)	−0.0031 (7)	0.0065 (7)	0.0030 (7)
C1	0.0233 (8)	0.0196 (8)	0.0192 (8)	0.0004 (7)	0.0036 (7)	−0.0026 (7)
C2	0.0347 (10)	0.0209 (8)	0.0199 (8)	−0.0056 (8)	0.0056 (8)	−0.0035 (7)
C3	0.0391 (11)	0.0267 (10)	0.0308 (10)	−0.0063 (8)	0.0126 (9)	−0.0034 (8)
C4	0.0523 (13)	0.0317 (10)	0.0342 (11)	−0.0115 (10)	0.0219 (10)	−0.0042 (9)
C5	0.0717 (16)	0.0323 (11)	0.0272 (10)	−0.0162 (11)	0.0190 (11)	−0.0001 (9)
C6	0.0593 (14)	0.0284 (10)	0.0228 (9)	−0.0058 (10)	0.0013 (9)	0.0015 (8)
C7	0.0434 (11)	0.0237 (9)	0.0191 (9)	−0.0057 (8)	0.0026 (8)	−0.0020 (7)
C8	0.0400 (11)	0.0293 (10)	0.0217 (9)	0.0011 (9)	−0.0065 (8)	0.0023 (8)
C9	0.0298 (10)	0.0303 (10)	0.0225 (9)	−0.0031 (8)	−0.0041 (8)	0.0001 (8)
C10	0.0217 (8)	0.0244 (9)	0.0205 (8)	−0.0004 (7)	−0.0006 (7)	−0.0016 (7)
C11	0.0175 (8)	0.0229 (8)	0.0231 (8)	0.0010 (7)	0.0037 (7)	−0.0042 (7)
C12	0.0174 (8)	0.0204 (8)	0.0205 (8)	−0.0001 (7)	0.0051 (7)	−0.0015 (7)
C13	0.0180 (8)	0.0185 (8)	0.0216 (8)	0.0019 (7)	0.0031 (7)	−0.0025 (7)
C14	0.0246 (9)	0.0218 (9)	0.0218 (9)	0.0016 (8)	0.0068 (8)	0.0016 (7)
C15	0.0161 (8)	0.0247 (9)	0.0243 (9)	−0.0003 (7)	0.0007 (7)	−0.0036 (7)
C16	0.0159 (8)	0.0251 (8)	0.0210 (8)	0.0015 (7)	−0.0006 (7)	0.0023 (7)
C17	0.0164 (8)	0.0281 (9)	0.0225 (8)	0.0019 (7)	0.0041 (7)	−0.0008 (7)
C18	0.0198 (8)	0.0219 (8)	0.0184 (8)	0.0010 (7)	0.0026 (7)	−0.0027 (7)
C19	0.0151 (8)	0.0210 (8)	0.0203 (8)	−0.0001 (6)	0.0013 (7)	0.0029 (7)
C20	0.0195 (8)	0.0294 (9)	0.0266 (9)	−0.0019 (7)	0.0072 (7)	−0.0068 (7)
C21	0.0230 (9)	0.0300 (10)	0.0247 (9)	−0.0023 (8)	0.0035 (7)	−0.0072 (8)
C22	0.0242 (10)	0.0402 (12)	0.0475 (12)	0.0015 (9)	0.0115 (9)	−0.0201 (10)
C23	0.0168 (8)	0.0287 (9)	0.0334 (10)	−0.0011 (7)	0.0079 (7)	−0.0061 (8)

### Geometric parameters (Å, °)

**Table:**

O1—C18	1.370 (2)	C8—H8A	0.9900
O1—C22	1.436 (2)	C8—H8B	0.9900
O2—C19	1.3690 (19)	C9—C10	1.512 (2)
O2—C23	1.434 (2)	C9—H9A	0.9900
N1—C1	1.397 (2)	C9—H9B	0.9900
N1—C13	1.411 (2)	C10—C11	1.391 (2)
N2—C14	1.153 (2)	C11—C12	1.400 (2)
N3—C13	1.351 (2)	C11—C16	1.494 (2)
N3—H1	0.95 (2)	C12—C13	1.413 (2)
N3—H2	0.92 (2)	C12—C15	1.436 (2)
N4—C15	1.150 (2)	C16—C21	1.374 (2)
C1—C10	1.413 (2)	C16—C17	1.399 (2)
C1—C2	1.484 (2)	C17—C18	1.383 (2)
C2—C3	1.396 (3)	C17—H17	0.9500
C2—C7	1.405 (3)	C18—C19	1.403 (2)
C3—C4	1.386 (3)	C19—C20	1.380 (2)
C3—H3	0.9500	C20—C21	1.394 (2)
C4—C5	1.381 (3)	C20—H20	0.9500
C4—H4	0.9500	C21—H21	0.9500
C5—C6	1.386 (3)	C22—H22A	0.9800
C5—H5	0.9500	C22—H22B	0.9800
C6—C7	1.392 (3)	C22—H22C	0.9800
C6—H6	0.9500	C23—H23A	0.9800
C7—C8	1.499 (3)	C23—H23B	0.9800
C8—C9	1.528 (3)	C23—H23C	0.9800
			
C18—O1—C22	115.94 (13)	C1—C10—C9	117.78 (15)
C19—O2—C23	116.23 (13)	C10—C11—C12	120.31 (15)
C1—N1—C13	121.85 (15)	C10—C11—C16	120.23 (15)
C13—N3—H1	121.0 (15)	C12—C11—C16	119.45 (15)
C13—N3—H2	121.2 (13)	C11—C12—C13	121.05 (15)
H1—N3—H2	117.7 (19)	C11—C12—C15	120.84 (14)
N1—C1—C10	119.15 (15)	C13—C12—C15	118.10 (14)
N1—C1—C2	122.66 (15)	N3—C13—N1	121.00 (15)
C10—C1—C2	118.16 (15)	N3—C13—C12	121.45 (16)
C3—C2—C7	119.44 (17)	N1—C13—C12	117.52 (14)
C3—C2—C1	122.73 (17)	N4—C15—C12	177.44 (18)
C7—C2—C1	117.76 (16)	C21—C16—C17	119.08 (15)
C4—C3—C2	120.60 (19)	C21—C16—C11	121.48 (15)
C4—C3—H3	119.7	C17—C16—C11	119.44 (15)
C2—C3—H3	119.7	C18—C17—C16	120.75 (15)
C5—C4—C3	119.7 (2)	C18—C17—H17	119.6
C5—C4—H4	120.1	C16—C17—H17	119.6
C3—C4—H4	120.1	O1—C18—C17	124.52 (15)
C4—C5—C6	120.47 (19)	O1—C18—C19	115.72 (14)
C4—C5—H5	119.8	C17—C18—C19	119.76 (15)
C6—C5—H5	119.8	O2—C19—C20	124.56 (15)
C5—C6—C7	120.5 (2)	O2—C19—C18	116.35 (14)
C5—C6—H6	119.7	C20—C19—C18	119.08 (15)
C7—C6—H6	119.7	C19—C20—C21	120.66 (16)
C6—C7—C2	119.19 (19)	C19—C20—H20	119.7
C6—C7—C8	122.56 (18)	C21—C20—H20	119.7
C2—C7—C8	118.19 (16)	C16—C21—C20	120.48 (16)
C7—C8—C9	108.73 (16)	C16—C21—H21	119.8
C7—C8—H8A	109.9	C20—C21—H21	119.8
C9—C8—H8A	109.9	O1—C22—H22A	109.5
C7—C8—H8B	109.9	O1—C22—H22B	109.5
C9—C8—H8B	109.9	H22A—C22—H22B	109.5
H8A—C8—H8B	108.3	O1—C22—H22C	109.5
C10—C9—C8	109.33 (14)	H22A—C22—H22C	109.5
C10—C9—H9A	109.8	H22B—C22—H22C	109.5
C8—C9—H9A	109.8	O2—C23—H23A	109.5
C10—C9—H9B	109.8	O2—C23—H23B	109.5
C8—C9—H9B	109.8	H23A—C23—H23B	109.5
H9A—C9—H9B	108.3	O2—C23—H23C	109.5
C11—C10—C1	119.86 (15)	H23A—C23—H23C	109.5
C11—C10—C9	122.36 (15)	H23B—C23—H23C	109.5

### Hydrogen-bond geometry (Å, °)

**Table:**

*D*—H···*A*	*D*—H	H···*A*	*D*···*A*	*D*—H···*A*
N3—H1···O1^i^	0.95 (2)	2.24 (2)	2.927 (2)	129 (2)
N3—H2···O2^i^	0.92 (2)	2.25 (2)	2.987 (2)	136 (2)

Symmetry codes: (i) *x*+1, −*y*+3/2, *z*+1/2.

## References

[bb1] Agilent (2010). *CrysAlis PRO* Agilent Technologies, Yarnton, England.

[bb2] Asiri, A. M., Al-Youbi, A. O., Faidallah, H. M. & Ng, S. W. (2011). *Acta Cryst.* E**67**, o2872. 10.1107/S1600536811040505 PMC3247607 22219912

[bb3] Barbour, L. J. (2001). *J. Supramol. Chem.* **1**, 189–191.

[bb4] Etter, M. C., MacDonald, J. C. & Bernstein, J. (1990). *Acta Cryst.* B**46**, 256–262. 10.1107/s0108768189012929 2344397

[bb5] Sheldrick, G. M. (2008). *Acta Cryst.* A**64**, 112–122. 10.1107/S0108767307043930 18156677

[bb6] Westrip, S. P. (2010). *J. Appl. Cryst.* **43**, 920–925.

